# Optical photothermal infrared spectroscopy can differentiate equine osteoarthritic plasma extracellular vesicles from healthy controls

**DOI:** 10.1039/d2ay00779g

**Published:** 2022-09-05

**Authors:** Emily J. Clarke, Cassio Lima, James R. Anderson, Catarina Castanheira, Alison Beckett, Victoria James, Jacob Hyett, Royston Goodacre, Mandy J. Peffers

**Affiliations:** Department of Musculoskeletal Biology and Ageing Science, Institute of Life Course and Medical Sciences, University of Liverpool William Henry Duncan Building, 6 W Derby St Liverpool L7 8TX UK eclarke@liverpool.ac.uk; Centre for Metabolomics Research, Biochemistry and Systems Biology, Institute of Systems, Molecular and Integrative Biology, University of Liverpool Biosciences Building, Crown Street Liverpool L69 7BE UK; Biomedical Electron Microscopy Unit, University of Liverpool UK; School of Veterinary Medicine and Science, University of Nottingham Sutton Bonington Loughborough LE12 5RD UK

## Abstract

Equine osteoarthritis is a chronic degenerative disease of the articular joint, characterised by cartilage degradation resulting in pain and reduced mobility and thus is a prominent equine welfare concern. Diagnosis is usually at a late stage through clinical examination and radiographic imaging, whilst treatment is symptomatic not curative. Extracellular vesicles are nanoparticles that are involved in intercellular communication. The objective of this study was to investigate the feasibility of Raman and Optical Photothermal Infrared Spectroscopies to detect osteoarthritis using plasma-derived extracellular vesicles, specifically differentiating extracellular vesicles in diseased and healthy controls within the parameters of the techniques used. Plasma samples were derived from thoroughbred racehorses. A total of 14 samples were selected (control; *n* = 6 and diseased; *n* = 8). Extracellular vesicles were isolated using differential ultracentrifugation and characterised using nanoparticle tracking analysis, transmission electron microscopy, and human tetraspanin chips. Samples were then analysed using combined Raman and Optical Photothermal Infrared Spectroscopies. Infrared spectra were collected between 950–1800 cm^−1^. Raman spectra had bands between the wavelengths of 900–1800 cm^−1^ analysed. Spectral data for both Raman and Optical Photothermal Infrared Spectroscopy were used to generate clustering *via* principal components analysis and classification models were generated using partial least squared discriminant analysis in order to characterize the techniques' ability to distinguish diseased samples. Optical Photothermal Infrared Spectroscopy could differentiate osteoarthritic extracellular vesicles from healthy with good classification (93.4% correct classification rate) whereas Raman displayed poor classification (correct classification rate = −64.3%). Inspection of the infrared spectra indicated that plasma-derived extracellular vesicles from osteoarthritic horses contained increased signal for proteins, lipids and nucleic acids. For the first time we demonstrated the ability to use optical photothermal infrared spectroscopy combined with Raman spectroscopy to interrogate extracellular vesicles and osteoarthritis-related samples. Optical Photothermal Infrared Spectroscopy was superior to Raman in this study, and could distinguish osteoarthritis samples, suggestive of its potential use diagnostically to identify osteoarthritis in equine patients. This study demonstrates the potential of Raman and Optical Photothermal Infrared Spectroscopy to be used as a future diagnostic tool in clinical practice, with the capacity to detect changes in extracellular vesicles from clinically derived samples.

## Abstract 2

This study demonstrates the potential of O-PTIR spectroscopy to be used as a future diagnostic tool in equine clinical practice, with the capacity to detect changes in extracellular vesicles from clinically derived equine osteoarthritis samples.

## Graphical abstract

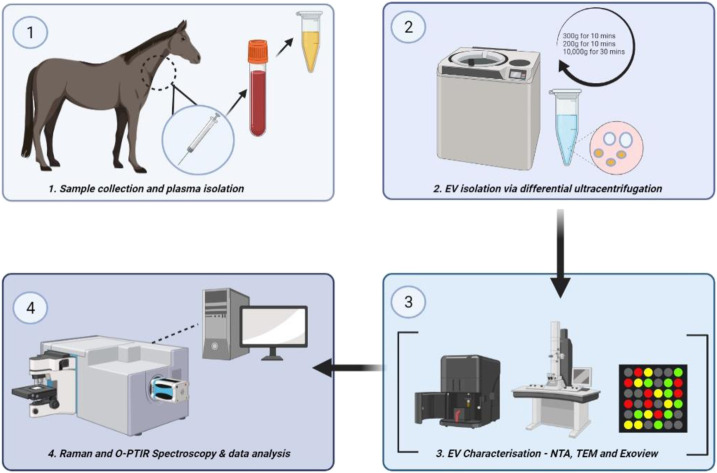

## Introduction

Osteoarthritis (OA) is a common degenerative disease of the synovial joint, characterised by catabolic processes observed in articular cartilage, and a notable imbalance in bone remodelling. It results in pain, inflammation and reduced mobility.^1^ OA is the most prevalent cause of equine lameness, with over 60% of horses developing OA within their lifetime; a significant welfare concern.^2^ It is a complex heterogeneous condition of multiple causative factors, including mechanical, genetic, metabolic and inflammatory pathway involvement.^3^ OA pathophysiology is conserved across species, resulting in synovitis, cartilage degradation, osteophyte formation, subchondral bone sclerosis, fibrosis and reduced elastoviscosity of synovial fluid found within the joint capsule.^4^ The horse is of interest not only as a target species for veterinary equine medicine, but also as a model organism to study osteoarthritis due to these established similarities, as well as comparable anatomic structures of the human carpal joint with equine carpal and metacarpophalangeal joints.^2^

Extracellular vesicles (EVs) are nanoparticles enveloped in a phospholipid bilayer membrane, secreted by most mammalian cells, that transport biologically active cargo, such as proteins, RNAs, DNAs, lipids, and metabolites.^5^ EVs are divided into subgroups determined by their size and biogenesis. EVs elicit their effects through paracrine signalling, proving fundamental in intercellular communication.^6^ EVs have been implicated in OA progression, and have been shown to be released and enter chondrocytes, synoviocytes and inflammatory cells.^7–9^ Interestingly EVs can serve as disease propagators; promoting an increased expression of cytokines, chemokines and matrix degrading proteinases, or disease preventing; increasing cellular differentiation and reducing apoptosis.^9^

As EVs are microscopic they require physicochemical characterisation and we consider that vibrational spectroscopic methods, including Raman and infrared spectroscopies, could provide valuable molecular information about the main molecular constituents commonly found in biological samples such as proteins, lipids, nucleic acids, and carbohydrates. This is based on functional group bond-specific chemical signatures being generated by these techniques in a non-invasive, non-destructive, and label-free manner.^10^ In Raman spectroscopy, photons from a monochromatic source interact with the sample and a small fraction of them are inelastically scattered with either higher or lower energies compared with the excitation wavelength. The energy difference between incident and scattered photons corresponds to a Raman shift and it is associated with the chemical structure of molecules in the sample.^11^ Raman spectroscopy can discriminate between cell and tissue types, and detect chemical alterations prior to morphological changes in various pathological states. It has previously been used to assess the purity of EV preparations,^12^ as well as identify cellular origin of mesenchymal stem cell (MSC)-derived EVs.^13^ Infrared spectroscopy is based on the absorption of infrared radiation by molecular vibrations from bonds that possess an electric dipole moment that can change by atomic displacement.^14^

Although Raman and infrared spectroscopies provide molecular information about the overall biochemistry of samples by monitoring the internal motion of atoms in molecules, each method has advantages and disadvantages for different types of samples due to their different working principles. Thus, having both techniques combined in a single platform is a powerful tool with promising applications. More recently, a novel far-field optical technique has been developed in order to acquire Raman and infrared signatures simultaneously from the same location on the sample. In this method, the infrared signatures are collected *via* Optical Photothermal Infrared Spectroscopy (O-PTIR), which is based on a pump-probe configuration that couples a tuneable infrared quantum cascade laser (QCL) acting as pump and a visible laser to probe the thermal expansion resulting from the temperature rise induced by the QCL. The probe laser also acts as excitation source for acquiring Raman spectrum simultaneously with infrared data at the same spatial resolution (*ca.* 500 nm spot diameter). This scheme has been used to interrogate tissue samples, mammalian cells^15,16^ and bacteria^17^ but this is the first study, to our knowledge, to use it in EV or OA studies.

We hypothesised that Raman and O-PTIR can be used to identify potential biomarkers of OA using equine plasma-derived EVs and aimed to test this using a combined vibrational spectroscopic platform.

## Materials and methods

### Sample selection

Plasma samples were collected in accordance with the Hong Kong Jockey Club owner consent regulations (VREC561). Samples were selected due to reflecting a natural model of post-traumatic osteoarthritis. All samples were from thoroughbred racehorses, and were collected between November 2005 and March 2009 from horses actively race training up to the time of death or had previously retired from active race training that were euthanized on welfare grounds. Samples were collected at post-mortem, within 30 minutes of death. The donor cohort had a mean age (± standard error of the mean (SEM)) of 6.57 ± 0.45. The same population has been used in previously published studies by Peffers *et al.* (2015).^18^ Horses were selected based on histological scoring of OA severity in the metacarpophalangeal joint using a modified Mankin score.^19^ In addition, osteoarthritis was clinically diagnosed by a qualified veterinary surgeon, to our knowledge these donors had no underlying co-morbidities. Synovitis scores were also obtained for donors, a total of 14 samples were selected (control; *n* = 6 (mean mankin score ± SEM = 1.83 ± 0.48), and mean synovitis score ± SEM = 3.7 ± 0.33) and diseased; *n* = 8 (mean mankin score ± SEM = 16.25 ± 1.15, and a mean synovitis score ± SEM = 5 ± 0.42), reflective of 14 equine donors.

### Histological scoring

Sections were obtained from the left medial distal metacarpal condyle and the left lateral distal metatarsal. Samples were cut with a band saw followed by a saline cooled diamond saw. Cartilage was subsequently dissected and underwent histological staining using Safranin O and Masson's trichrome in order to score cartilage pathologic lesions using a modified Mankin score. This score evaluates cartilage erosion, chondrocyte periphery staining, spatial arrangement of chondrocytes, and background staining intensity. With a higher value denoting a more severe OA phenotype in addition, the synovial membranes of metacarpophalangeal joints were harvested postmortem and fixed in cold 4% formaldehyde for at least 30 min before being processed. Processing involved paraffin-embedding, and 10 μm section cutting. Synovium sections were hematoxylin and eosin-stained to assess the level of synovitis, and was determined by three components of synovitis: lining layer hyperplasia, activation of resident cells (stroma) and inflammatory infiltrate. Whereby a higher score denotes a greater severity of synovitis.

### Extracellular vesicle isolation – differential ultracentrifugation

Equine plasma samples underwent differential ultracentrifugation (dUC) in order to isolate EVs. Samples were subjected to a 300×*g* spin for 10 min, 2000×*g* spin for 10 min, 10 000×*g* spin for 30 min in a bench top centrifuge at room temperature. Samples were then transferred to Beckman Coulter thick wall polycarbonate 4 mL ultracentrifugation tubes, and spun at 100 000×*g* for 70 min at 4 °C (Optima XPN-80 Ultracentrifuge, Beckman Coulter, California, USA) in a 45ti fixed angle rotor, with the use of a 13 mm diameter Delrin adaptor. Sample pellets were suspended in 50 μL of filtered phosphate buffered saline (PBS) (Gibco™ PBS, pH 7.4 – Fisher Scientific, Massachusetts, USA), resulting in 50 μL plasma EV (P-EV) samples.

### Extracellular vesicle characterisation

#### Nanoparticle tracking analysis

Nanoparticle tracking analysis (NTA) was used to quantify EV concentration and size of all samples, using a NanoSight NS300 (Malvern Panalytical, Malvern, UK). All samples were diluted in filtered PBS 1 : 50 (10 μL of sample used), to a final volume of 500 μL. For each measurement, three 1 min videos were captured, (at a screen gain of 4 and detection threshold of 12). After capture, the videos were analysed by the in-build NanoSight Software NTA 3.1 Build 3.1.46. Hardware: embedded laser: 45 mW at 488 nm; camera: sCMOS.

#### Transmission electron microscopy

EV presence and morphology were characterised using transmission electron microscopy (TEM). 10 μL of each sample was placed onto a carbon coated glow discharged grid and incubated at room temperature for 20 min. Samples were then subject to a negative staining protocol. EVs were fixed onto the grid with 1% glutaraldehyde for 5 min. The sample grids were incubated on 1% aqueous uranyl acetate (UA) (Thermofisher Scientific, Massachusetts, USA), for 60 s, followed by 4% UA/2% methyl cellulose (Sigma Aldrich, Gillingham, UK) at a 1 : 9 ratio on ice for 10 min. Grids were then removed with a 5 mm wire loop and dried. The prepared grids were then viewed at 120 KV on a FEI Tecnai G2 Spirit with Gatan RIO16 digital camera.

#### ExoView characterisation

The ExoView platform (NanoView Biosciences, Malvern Hills Science Park, Malvern) was used to determine EV concentration, surface marker identification and to perform fluorescent microscopy and tetraspanin colocalisation analysis. We had previously tested both the human and murine chips on equine samples and demonstrated the human chips were more compatible (data not shown). ExoView analyses EVs using visible light interference for size measurements and fluorescence for protein profiling. Samples were analysed in triplicate using the ExoView Tetraspanin Kit (NanoView Biosciences, USA) and were incubated on the human ExoView Tetraspanin Microarray Chip for 16 h at room temperature. Following this sample chips were incubated with tetraspanin labelling antibodies, namely anti-CD9 CF488, anti-CD81 CF555 and anti-CD63 CF647 and the MIgG negative control. The antibodies were diluted 1 : 500 in PBS with 2% bovine serum albumin. The chips were incubated with 250 μL of the labelling solution for 1 h. The sample chips were washed and imaged with the ExoView R100 reader ExoView Scanner v3.0. Data were analysed using ExoView Analyzer v3.0. Fluorescent cut offs were set relative to the MIgG control. Total EVs were determined as the number of detected particles bound to tetraspanin antibodies (CD9, CD81, CD63) and normalised to MIgG antibody.

#### Raman spectroscopy and infrared (O-PTIR) spectroscopy

For all samples O-PTIR measurements were acquired on single-point mode using a mIRage infrared microscope (Photothermal Spectroscopy Corp., Santa Barbara, USA), with the pump consisting of a tuneable four-stage QCL device, while the probe beam is a continuous wave 532 nm laser. Spectral data were collected in reflection mode using a 40×, 0.78 NA, and 8 mm working distance Schwarzschild objective. A total number of 130 single-point infrared spectra (10 spectra per sample/horse) were acquired over a spectral region of 930–1800 cm^−1^, with 2 cm^−1^ spectral resolution and 10 scans per spectrum. 130 Raman spectra (10 spectra per sample/horse) were acquired simultaneously with infrared data using a Horiba Scientific iHR-320 spectrometer coupled to mIRage, using a grating of 600 L mm^−1^, 10 s as acquisition time, spectral region of 500–3400 cm^−1^, with 2 cm^−1^ spectral resolution and 10 scans per spectrum. It should be highlighted that the volumes used for all samples were the same for both O-PTIR and Raman spectroscopy measurements, samples were not diluted for this analysis.

#### Statistical analysis

Raman and O-PTIR spectroscopic data were analysed using principal component analysis (PCA) to determine each techniques ability to identify diseased samples from healthy controls. Before PCA and PLS-DA, spectra were submitted to baseline correction using an asymmetric least squares algorithm, smoothed *via* a Savitzky–Golay filter, and vector normalized. All spectral data were used as input. Spectral data were also used as input for partial least squares discriminant analysis (PLS-DA) in order to generate a classification model for differentiation of OA from controls. Further analysis with PLS-DA involved using a classification model and confusion matrices, whereby resampling of the data involved both bootstrapping 10 000 times with permutation testing to generate null models to assess whether an EV spectrum was classified as OA or control.

## Results

### Statistical evaluation of histological and synovitis scores

For statistical evaluation of histological scoring parametric *T*-test was used. For evaluation of synovitis score non-parametric Mann–Whitney *U* test was used. Statistical evaluation was performed between control and diseased using GraphPad Prism 8 (San Diego). Histological score was significantly different between groups (*p* < 0.0001). Synovitis score between groups was not significant (*p* = 0.12).

### Nanoparticle tracking analysis

**Fig. 1:**
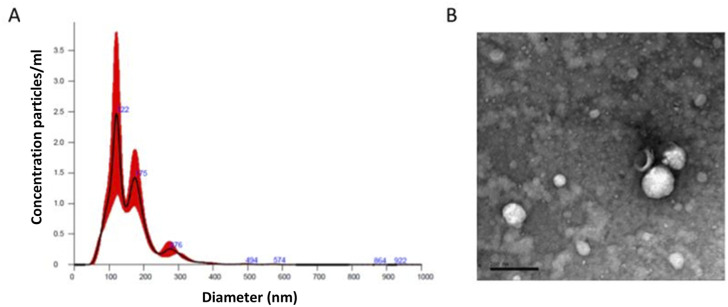
Characterisation of EVs. (A) Nanoparticle tracking analysis (NTA) of a representative sample of plasma derived EVs (P-EVs). Concentrations of EVs in particles per mL and particle size measured in nm, all measurements recorded using NanoSight NS300, and data analysed by the in-build NanoSight Software NTA 3.1 Build 3.1.46. Hardware: embedded laser: 45 mW at 488 nm; camera: sCMOS. (B) Transmission electron microscopy (TEM) micrograph of negatively stained representative of plasma derived EV samples. Scale bar is equal to 200 nm. Samples fixed to grids were visualised using a FEI Tecnai G2 Spirit with Gatan RIO16 digital camera.

Particle size and concentration characterisation was performed using NTA. NTA determined the average plasma sample concentration to be 2.02 × 10^9^ particles per mL. Analysis was suggestive of a heterogeneous population of EVs, ranging from exosomes to microvesicles (Fig. 1A).

### Transmission electron microscopy

To confirm the particles isolated from plasma samples were indeed EVs we negatively stained and visualised them using TEM. Spherical structures within EV size ranges (30 nm–100 nm (exosomes)) and 100 nm–1000 nm (microvesicles) were identified with a clearly defined peripheral membrane as shown in Fig. 1B.

### Exoview

**Fig. 2:**
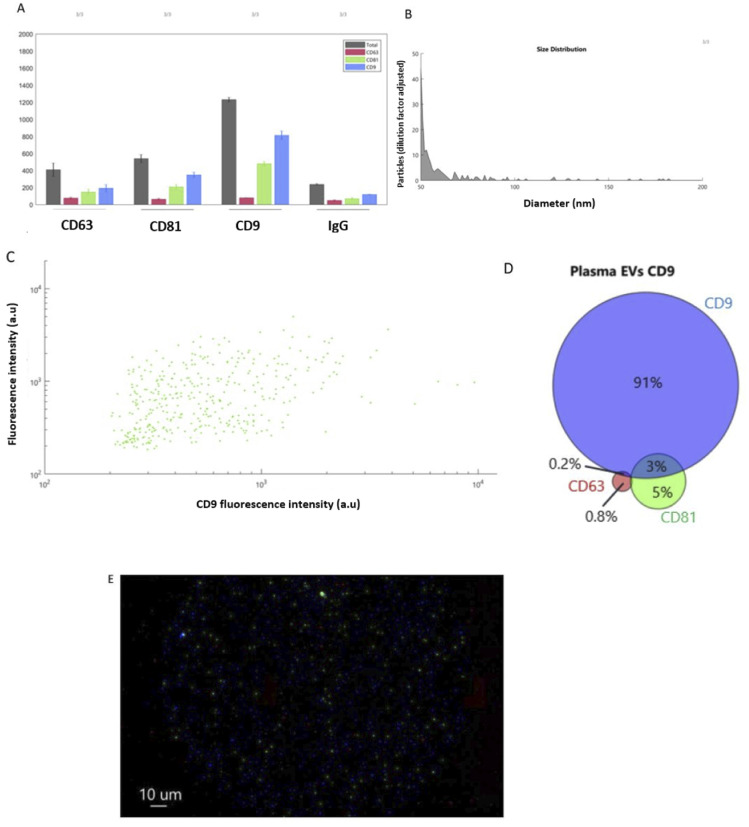
ExoView assay results. (A) Particle counts for plasma EVs from the human tetraspanin chip, (B) a size histogram of plasma EV samples as captured on the CD9 human tetraspanin chip, (C) a scatter diagram demonstrating correlation between the number of CD9 positive EVs (*X* axis) and CD81 positive EVs (*Y* axis), (D) colocalization analysis of the presence of surface tetraspanins on equine plasma EVs, and (E) fluorescent microscopy visualising plasma-derived EVs, with colour denoting surface tetraspanin positive identification (red – CD63, blue – CD9, and green – CD81).

ExoView was used on a representative pool of plasma samples. The EVs isolated from plasma had the highest particle counts on the CD9 capture spots, equating to a concentration of approximately 1.4 × 10^8^ CD9 positive particles per mL. CD81 (7 × 10^7^ particles per mL) and CD63 (6 × 10^7^ particles per mL positive particles were also detectable (Fig. 2A)). It was observed that most EVs detected were less than 100 nm (Fig. 2B). It was also found that with plasma derived EV samples, the greater the expression of CD9 the greater the corresponding expression of CD81, whereby a distinct positive correlation was observed (Fig. 2C). Co-localisation analysis was also performed and identifying that 91% of plasma-derived EVs were positive for the CD9 surface tetraspanin, followed by 5% expressing CD81, and 3% expressing both CD81 and CD9. CD63 expression was lowest at 0.8% (Fig. 2D). Finally, EVs were visualised using fluorescent microscopy, as shown in Fig. 2E.

### Raman spectroscopy and O-PTIR spectroscopy

**Fig. 3:**
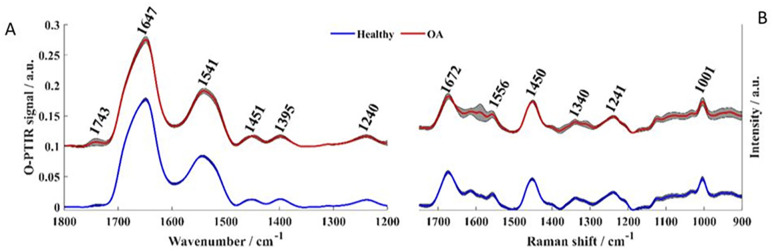
Fingerprint region of averaged spectra. (A) Infrared and (B) Raman spectra collected from healthy control (blue line) and OA (red line) samples. Plots are offset for clarity.

Infrared and Raman spectral data collected from healthy control and diseased plasma derived EV samples displayed similar biochemical features with subtle changes in signal intensity, while appearance or disappearance of peaks were not observed (Fig. 3A and B).

Infrared signatures acquired by O-PTIR spectroscopy were recorded from 950–1800 cm^−1^ as this is the spectral range covered by the tunable QCL used as excitation source, however, bands peaking below 1200 cm^−1^ were removed from the analysis due to interference from bands attributed to minerals from PBS.^20^ The band peaking at 1743 cm^−1^ arose from C=O ester groups from lipids including phospholipids, triglycerides and cholesterol^20^ Amide I vibration, peaking at 1647 cm^−1^ was associated mainly to C=O stretching vibration from peptide bonds in proteins.^20–22^ Amide II band absorption was found in 1544 cm^−1^ and is attributed to the out-of-phase combination of the N–H in-plane bend and C–N stretching vibration with smaller contributions from the C–O in-plane bend and the C–C and N–C stretching vibrations of peptide groups.^20–22^ The band observed at 1240 cm^−1^ results from the coupling between C–N and N–H stretching from proteins (amide III),^21^ but it was also influenced by PO_2_-asymmetric stretching from phosphodiester bonds in nucleic acids [10]. The peak at 1451 cm^−1^ corresponded to bending vibration (scissoring) of acyl CH_2_ groups in lipids,^20–22^ whereas the band peaking at 1395 cm^−1^ arises from COO– symmetric stretching from amino acid side chains and fatty acids.^20–22^ Although Raman signatures were collected between 500–3400 cm^−1^, only the fingerprint region (500–1800 cm^−1^) was evaluated as the vast majority of molecular vibrations are found peaking in this region. Spectral signatures from minerals were also observed in Raman spectrum acquired from healthy and diseased samples in the low wavenumber region (below 900 cm^−1^), therefore, only bands peaking between 900–1800 cm^−1^ were analysed. In Raman spectra, peaks associated with amide I, II, and III from peptide bonds were observed peaking at 1672, 1556, and 1241 cm^−1^ respectively.^12,23^ The band at 1450 cm^−1^ originated from CH_2_/CH_3_ bending vibrations from lipids and proteins, while the peak at 1004 cm^−1^ was attributed to the phenylalanine ring breathing, and the peak at 1340 cm^−1^ was associated to nucleic acids.^12,23^

**Fig. 4:**
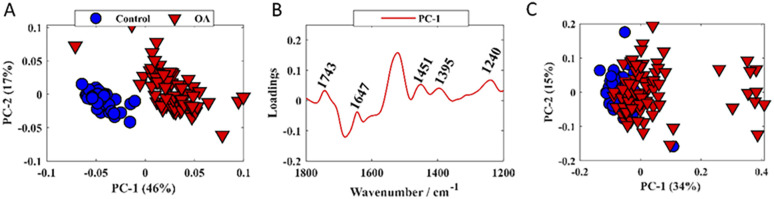
PCA scores and loadings plots. Plots were obtained by subjecting infrared data to PCA (A and B); (C) PC scores plot of Raman data; values in parentheses are the percentage total explained variance (TEV).

Infrared and Raman were first subjected to principal component analysis (PCA) in order to examine the ability of both techniques to discriminate healthy control and diseased plasma-derived EV samples (Fig. 4).

PC scores plot obtained from O-PTIR (infrared signatures) as input data showed satisfactory discrimination between healthy control and diseased samples (Fig. 4A), with scores from control samples grouping on the negative side of PC-1 axis, whilst scores related to OA clustered on positive side of the PC-1 axis. The loadings plot (Fig. 4B) was used to reveal the importance of the input variables on the PCA and positive loadings to all bands displayed in Fig. 4A, indicating higher amount of the molecular constituents associated with these vibrations, *i.e.*, proteins, lipids and nucleic acids, in samples derived from plasma EVs in OA. The scores plot obtained by subjecting Raman signatures to PCA displayed poor discrimination between healthy control and OA samples (Fig. 4C). This was why we did not display the corresponding loadings plot.

**Fig. 5:**
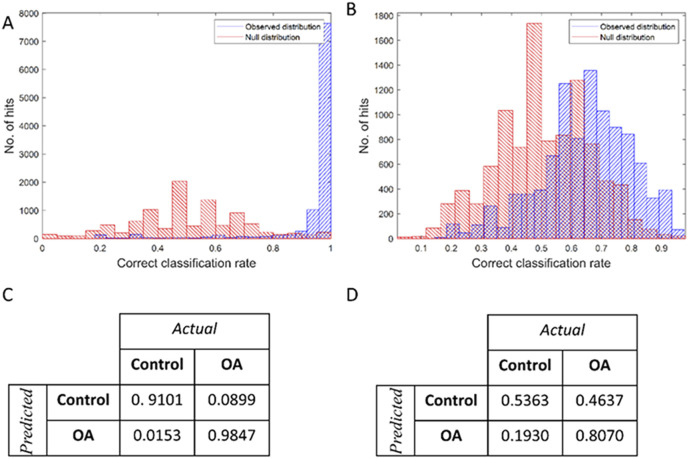
Classification model for PLS-DA of infrared and Raman spectra. (A) Infrared and (B) Raman spectra showing classification rates for real (blue – from the 10 000 bootstrap analyses) and random (red – from the 10 000 permutation tests), with a sensitivity, specificity, and positive predictive value of 97.5%, 97.6%, 96.7% for infrared, respectively. (C) and (D) are the average confusion matrices for infrared and Raman, respectively, with rows representing predicted classification and columns representing experimental.

Raman and O-PTIR spectral data were used as input for PLS-DA in order to generate a model for classifying OA and control samples based on EV composition. In this study, a total of 130 spectra (10 spectra per animal; 50 spectra from healthy and 80 spectra from OA) were used as input data for PLS-DA. Each IR spectrum has 300 variables (O-PTIR intensities associated to each wavenumber between 1200–1800 cm^−1^), whereas each Raman spectrum provides 480 variables. The classification models and confusion matrices obtained by PLS-DA using O-PTIR and Raman data are shown in Fig. 5. Bootstrapping and permutation testing were performed 10 000 times to assess overall model performance and to decide whether the EV spectrum was classified as OA or control. PLS-DA model obtained from O-PTIR data achieved of sensitivity, specificity, and positive predictive values of 97.5%, 97.6%, 96.7%, while PLS-DA model generated by using Raman signatures as input data presented poor classification rates (sensitivity (66.37%), specificity (66.37%), and positive predictive values (64.62%)). These findings agree with the results obtained by PCA, indicating superior ability of O-PTIR to discriminate healthy from OA samples.

## Discussion

This study investigated for the first time whether O-PTIR and Raman spectroscopies could be used successfully to interrogate plasma-derived EVs in control and OA equine samples. We demonstrated that indeed the novel spectroscopy technique O-PTIR was able to determine differences in EV content attributed to disease pathology, while Raman spectroscopy provided poorer classification models. Thus O-PTIR could serve as a platform for as future biomarker studies in equine OA.

Clinical equine OA samples were used due to an interest in exploring the EV metabolome of equine OA. The fetlock joints were assessed for OA by histological scoring at post-mortem. The fetlock joint (metacarpophalangeal joint) is the most commonly afflicted joints in equine athletes.^24^ Thoroughbred race horses are known to be a valuable model of natural occurring post traumatic osteoarthritis, with an increased prevalence of disease as a result of sustained repetitive impact, leading to subsequent joint injuries.^25^ As such our sample population was used as an appropriately characterised set of OA samples, with adequate volume to isolate EVs and use the proposed techniques to answer our research question. Additionally, we had extensive clinical records for our sample population. Finally, all horses were stabled, managed and raced at the same location enabling horses exposed to similar husbandry to be used.

Mankin score between control and diseased was significantly different (*p* < 0.0001). However, synovitis score between groups was not (*p* = 0.12), this may be reflective of the equine discipline the sample donors were used within, as horseracing involves a repetitive motion and large forces being applied to joints in the associated limbs, subsequently resulting in a degree of trauma to the joint capsule. In humans OA is often accompanied by inflammation caused by the immune response.^26^ However, we have described that there is no correlation between OA and synovitis score in horses with metacarpophalangeal OA.^27^ Whilst we had extensive clinical and post-mortem records that did not indicate the horses had other underlying inflammatory conditions without additional studies in other inflammatory conditions we cannot rule out that changes were not due to inflammation as opposed to OA. Thus OPTIR may be differentiating other factors not exclusive to OA.

EVs are a heterogenous group of nanoparticles, categorized based on size and biogenesis, however it is common practice to design experiments encompassing microvesicles and exosomes, due to the current inability of techniques to confidently isolate specific subgroups alone. This is reflective of the infancy of this research field. It is known that choice of isolation method can alter the heterogeneity of EV sample collected, with varying proportions of vesicles derived from both endosomal multivesicular bodies and plasma membrane budding as a result. The most common isolation methods include size exclusion chromatography, precipitation and differential ultracentrifugation. As demonstrated by Brennan *et al.*^28^ in a study comparing human serum EVs, noting the number of EVs, protein and lipoprotein content varied between isolation method.

Plasma EV concentration (2.02 × 10^9^ particles per mL) was quantified using NTA, an accepted method of EV evaluation known for its repeatability and reproducibility.^29^ The representative plasma sample shown reflects the biological sample heterogeneity, showing a range of EV sizes indicative of both exosome and microvesicles subgroups. The concentration determined was similar to those reported for plasma EVs in other species within the literature. For example, human plasma EVs identified by Palviainen *et al.*^30^ 2.46 × 10^9^–1.10 × 10^10^ particles per mL. However, a study analysing plasma EVs across the duration of equine endurance racing found that baseline plasma EV concentration was 5.6 × 10^12^ particle per mL.^31^ The difference observed may be a result of horse breed and discipline used within. ExoView analysis identified EVs that were positive for the tetraspanins CD81 and CD9, however a low percentage of EVs were positive for the surface marker CD63. This may a result of poor protein homology between equine and humans for this protein. However, a recent paper using intracellular trafficking demonstrated that CD63 expression in HeLa cells was specific to exosomes, and often a lack of CD63 expression may be due to small microvesicle production, referred to as ectosomes, and that this type of EV is far more prolific than CD63 positive exosomes.^32^

PCA and PLS-DA were used to analyse spectral data in order to evaluate the outputs obtained by an unsupervised (PCA) and a supervised (PLS-DA) method. PCA is the gold standard for unsupervised analyses and highlights that EVs can be separated using O-PTIR but not from Raman data. PLS-DA is a popular method applied to metabolomics and spectroscopy for supervised learning and hence PLS-DA was used to corroborate the PCA results. Infrared spectroscopy techniques have been used to probe EVs previously in order to identify structural components^33^ as well as proteins, lipid and nucleic acid components, as found in our study.^33^ This is the first paper to our knowledge to probe OA EVs using Raman spectroscopy and O-PTIR spectroscopy which have the advantage that they probe a very small volume. In other work, Zhai *et al.*^34^ found that in bone, mineral and carbonate content varied significantly with OA stage, with carbonate increasing with OA. They also identified using Fourier transform infrared (FT-IR) spectroscopy that acid phosphate, collagen maturity and crystallinity varied with OA. In addition, the use of infrared spectroscopy is compounded by the findings of Afara *et al.*^35^ in a study utilising an experimental model of OA in rats. Here the spectral differences between control and OA samples could be correlated to Mankin score and glycosaminoglycan content. A previous study by our group used attenuated total reflection FT-IR (ATR-FTIR) spectroscopy on OA equine serum. This infrared spectroscopy study found separation between groups with 100% sensitivity and specificity, and the six most significant peaks between groups was attributed to proteins and lipids. Similarly, this was observed within our current study, with increased abundance found within the OA group. The stated study postulated these observations may be associated to increased lipid and protein expression including increased expression of type 1 collagen, and decreased expression of type 2 collagen characteristic of OA.^36^

Previously, changes in plasma and lipid concentration in plasma and serum derived from OA patients has been described. One study utilised serum samples from horses to discriminate proteomic changes due to exercise or the development of early OA.^37^ Researchers identified six biomarkers with the ability to discriminate OA from exercise groups. For example, the concentration of serum C1, 2C (reflective of type 1 and 2 collagen degradation fragments) and collagen 1 was found to significantly increase in OA groups compared with exercise alone.^37^ In addition, a multiplexed proteomic study on human OA serum identified a panel of 14 candidate biomarkers for OA,^38^ utilising cartilage, synovial fluid, chondrocytes and serum. These prospective biomarkers include von Willebrand factor (inflammation and haemostasis) and haptoglobin (an inflammation inducible plasma protein).^38^ Furthermore, a previous study in plasma identified different lipid profiles in OA using a destabilisation of the medial meniscus model in mice.^39^ Altered lipids included classes of cholesterol esters, fatty acids, phosphatidylcholines, *N*-acylethanolamines, and sphingomyelins and some were attributed to cartilage degradation.

Raman spectroscopy has been employed to characterise EVs and their composition. It has been used to interrogate EVs undergoing autophagy,^40^ as well as distinguishing EVs derived from bovine placenta and mononuclear cells,^23^ identifying differential features in Parkinson's disease pathology^41^ and sporadic Amyotrophic Lateral Sclerosis.^42^ Additionally, it has been applied to successfully discriminate between healthy and diseased joint tissues in order to identify subtle molecular and biochemical changes as a result of disease. Buchwald *et al.*^43^ utilised Raman spectroscopy to identify compositional and structural changes in bone from the hip joints of OA patients demonstrating that subchondral bone from OA patients was less mineralised due to a decrease in hydroxyapatite. Furthermore, a study performed by de Souza *et al.*^44^ using two *in vivo* experimental rat models of knee OA (treadmill exercise induced and collagenase induced) established molecular signatures unique to OA. Raman ratios relating to mineralization and tissue remodelling were significantly higher in OA groups. Specifically, the ratio between phosphate and amide III has been shown to reflect the degree of mineralisation and carbonate/amide III is indicative of bone remodelling. De Souza *et al.*^44^ also commented on the lack of literature available with regard to the use of Raman spectroscopy for OA research and the importance of this developing field. More recently, Hosu *et al.*^45^ reviewed the importance of Raman spectroscopy in identifying pathologically associated crystals such as monosodium urate and calcium pyrophosphate dihydrate in rheumatoid diseases.

Optical photothermal infrared spectroscopy requires minimal sample preparation enabling reduced analysis time when obtaining submicron spatial resolution.^46^ Subsequently O-PTIR is advantageous over other traditionally used methods such as Fourier transform infrared spectroscopy and quantum cascade laser microscopy.^47^ This makes it a useful technique as it is also highly sensitive. Furthermore, the equipment used is smaller in size compared to other metabolomic techniques, such as nuclear magnetic resonance spectroscopy. Thus, having the possibility of placement ‘stableside’ or with an equine diagnostic laboratory. As such it has a greater capacity to be used in a clinical setting in the future. One downside is that currently O-PTIR analysis of EV samples requires concentration in order to achieve an appropriate signal.

We recognise a number of limitations in our study. We were restrained by the number of samples available to us, and our findings need validation in a larger cohort. Our sample was small due to the use of clinically sourced samples. Future studies will require replication of our analysis with a large cohort of samples. There are currently no definitive equine OA EV markers. Therefore, we cannot say definitively that the plasma-derived EVs used in this paper are from the OA joint or due to OA. Thus we cannot say definitively that OPTIR is not differentiating other factors not exclusive to OA. We are currently undertaking a number of studies to identify equine EV markers specific for OA including mass spectrometry lipidomic, NMR lipidomic and small non-coding RNA sequencing studies of equine EVs. In our recent paper^48^ we described a number of small non-coding RNAs changing in OA in both plasma and synovial fluid derived EVs. Unfortunately, we did not have any remaining plasma from the horses used in this study to complement our findings here and show the EVs interrogated were OA related. Thus, in the future additional studies will be able to determine if the EVs isolated in plasma in this study are OA specific. In addition, large sample volumes were necessary to have an adequate number of EVs for analysis, providing an appropriate signal to noise ratio. Moreover, due to the large sample volumes required repeated studies using other appropriate techniques such as NMR spectroscopy for validation were not possible. In our future studies minimum sample volume required will be optimized. Additionally, we used a single time point ‘snap shot’ of disease. Further work is needed to determine if O-PTIR is sensitive enough to determine differences in a range of different OA phenotypes and severities, and correlate differences to specific biological functions of EVs.

Overall this study demonstrates a ‘proof of concept’ with respect to the potential of Raman and OPTIR spectroscopy to be used as a diagnostic tool in clinical practice. Further work is required to identify if OA-related changes in plasma-derived EVs is related to the pathogenesis in the joint by utilising mechanistic study design, as well as exploring further validation with the use of additional metabolomics techniques-such as NMR metabolomics. We are currently quantifying the EV cargo using NMR metabolomics, mass spectrometry proteomics and sequencing platforms in order to provide complete characterisation of EVs in OA and determine their contribution to disease propagation.

## Conclusions

In conclusion, EVs derived from equine plasma in OA were probed using Raman and O-PTIR spectroscopy. O-PTIR spectroscopic analysis was found to be superior in classifying samples from OA patients compared to Raman spectroscopy. O-PTIR spectroscopy is an exciting potential platform for the analysis of plasma to diagnose OA-in both human and horse, with this study pathing the way for future research to explore the spectroscopic techniques diagnostic capacity further.

## Author contributions

Conceptualization, M. J. P., R. G., C. L., and E. J. C.; methodology, E. J. C., M. P., R. G., C. L., C. C., J. A., J. H., V. J., A. B.; formal analysis, C. L. and E. J. C.; investigation, C. L. and E. J. C.; data curation, C. L. and E. J. C.; writing—original draft preparation, E. J. C.; writing—review and editing, E. J. C., C. L., M. P., R. G., C. C., J. A., V. J., J. H., A. B.; visualization, C. L. and E. J. C.; all authors have read and agreed to the published version of the manuscript.

## Conflicts of interest

The authors declare no conflict of interest.

## Supplementary Material
